# Mitochondrial DNA 7908–8816 region mutations in maternally inherited essential hypertensive subjects in China

**DOI:** 10.1186/s12920-018-0408-0

**Published:** 2018-10-16

**Authors:** Ye Zhu, Xiang Gu, Chao Xu

**Affiliations:** 1 Clinical Medical College, Yangzhou University, Yangzhou, 225001 Jiangsu China; 2 Department of Cardiology, Northern Jiangsu People’s Hospital, Yangzhou, 225001 Jiangsu China; 3 Department of Biostatistics and Epidemiology, University of Oklahoma Health Science Center, Oklahoma City, OK 73134 USA

**Keywords:** Mitochondria, DNA, Mutation, Essential hypertension, Maternal inheritance

## Abstract

**Background:**

Nuclear genes or family-based mitochondrial screening have been the focus of genetic studies into essential hypertension. Studies into the role of mitochondria in sporadic Chinese hypertensives are lacking. The objective of the study was to explore the relationship between mitochondrial DNA (mtDNA) variations and the development of maternally inherited essential hypertension (MIEH) in China.

**Methods:**

Yangzhou residents who were outpatients or in-patients at the Department of Cardiology in Northern Jiangsu People’s Hospital (Jiangsu, China) from June 2009 to June 2015 were recruited in a 1:1 case control study of 600 gender-matched Chinese MIEH subjects and controls. Genomic DNA was isolated from whole blood cells. The most likely sites for hypertension were screened using oligodeoxynucleotides at positions 7908–8816, purified and subsequently analyzed by direct sequencing according to the revised consensus Cambridge sequence. The frequency, density, type and conservative evolution of mtDNA variations were comprehensively analyzed.

**Results:**

We found a statistical difference between the two groups for body mass index, waist circumference, abdominal circumference, triglyceride, low-density lipoprotein cholesterol, fasting blood glucose, uric acid, creatinine and blood urea nitrogen (*P* < 0.05). More amino-acid changes and RNA variants were found in MIEH subjects than the controls (*P* < 0.01). The detection system simultaneously identified 40 different heteroplasmic or homoplasmic mutations in 4 genes: COXII, tRNA^Lys^, ATP8 and ATP 6. The mtDNA variations were mainly distributed in regions of ATP6 binding sites, and the site of highest mutation frequency was m. 8414C > T. Three changes in single bases (C8414T in ATP8, A8701G in ATP6 and G8584A in ATP6) were significantly different in the MIEH patients and the controls (*P* < 0.001). The m.8273_8281del mutation was identified from 59 MIEH patients.

**Conclusions:**

Our results indicate that novel mtDNA mutations may be involved in the pathological process of MIEH, and mitochondrial genetic characteristics were identified in MIEH individuals.

**Electronic supplementary material:**

The online version of this article (10.1186/s12920-018-0408-0) contains supplementary material, which is available to authorized users.

## Background

Essential hypertension (EH) is a common chronic disease which is becoming an urgent public health issue worldwide, accounting for 9.4 million deaths each year [1]. EH is characterized by an elevation in arterial pressure and is a major risk factor for many common causes of morbidity and mortality including myocardial infarction, congestive heart failure, stroke and kidney failure in many segments of the population [2]. EH results from the interaction between environmental and inherited risk factors, which can be caused by single-gene or multifactorial conditions.

A family history of hypertension means that individuals are more likely to suffer hypertension [3, 4]. Maternally inherited essential hypertension (MIEH) is EH that shows a pattern of maternal inheritance and is occasionally observed in the clinic [5]. mtDNA can lead to mitochondrial diseases that are exclusively transmitted from the mother. mtDNA mutations have been identified in some pathogenic diseases such as myoclonic epilepsy, lactic acidosis, mitochondrial myopathy, encephalopathy, stroke-like episodes, and ragged-red fibers or maternally inherited diabetes [6]. Accordingly, mutations in mtDNA have also been reported in MIEH [7].

Mitochondria have an inefficient DNA repair and protection system in comparison to that of nuclear DNA [8]. All the homoplasmic mtDNA mutations that have been identified as being related to MIEH have caused functional disorders. The m.4435A > G mutation that is located immediately at the 3’end of the anticodon. This location corresponds to position 37 of tRNAMet affecting codon recognition, structural formation, and stabilization of functional tRNAs [9]. The m.4263A > G mutation reduces the efficiency of the tRNAIle precursor 5′-end cleavage that is catalyzed by RNase P because it is located at the processing site for the tRNAIle 5′-end precursor [10]. The result of these mutations is abnormal mitochondrial respiration that causes oxidative stress, this uncouples the oxidative pathways for ATP synthesis, and leads to cellular energetic processes failing [11].

To date, the roles of somatic mtDNA mutations in MIEH are still poorly understood. The development of blood pressure and this increases with many factors that include the mtDNA mutation/background, nuclear genes and environmental factors [12]. There is some suggestion that gene variations are associated with hypertension; but different results have been seen in different populations [13]. The mitochondrial genome accounts for ~ 5% of the heritability of blood pressure and these increases to ~ 35% for hypertensive pedigrees [14, 15]. With improved genetic analysis techniques, in particular genome-wide association studies, genes can now be identified that are likely to contribute to the development of hypertension within populations [16]. However, most genetic mutations have been identified in the nuclear genome [17]; only a few studies have focused on the investigation of the mitochondrial genome in the development of hypertension in populations. Therefore, it is obvious that understanding mtDNA sequence alteration involvement in MIEH may improve understanding of the genetic basis and pathogenesis of MIEH.

MtDNA mutations mainly distributed in the 7908–8816 region as described previously [18]. In this study, to understand more about the molecular mechanism underlying MIEH, we undertook screening of study in the mtDNA 7908–8816 region in hypertensive and normotensive subjects in a systematic and extensive manner. We explored inherited and clinical evidence to observe the relationship between the mitochondrial genome and MIEH. We decided to focus upon a Chinese Han population, as the morbidity of EH in Chinese adults is nearly 11.8% [19] but there is a limited amount of study on this racial group.

## Methods

### Subjects

This was a case control study of 300 unrelated patients with MIEH and 300 healthy control subjects. The MIEH participants were selected according to the following inclusion criteria: (1) outpatients or in-patients underwent a regular medical check-up at the Department of Cardiology in Northern Jiangsu People’s Hospital from June 2009 to June 2015; (2) more than 18 years old; (3) with a diagnosis of primary hypertension; (4) not receiving antihypertensive medication; (5) diagnosed with MIEH according to the maternal transmission of EH within generations, which was transmitted by the mother or her relatives and not by the father. Patients were excluded if they were diagnosed with: (1) secondary hypertension (for example renal arterial sterosis, hyperaldosteronism, aortic coarctation, and pheochromocytoma); (2) congenital heart diseases; and (3) presence of organic valve diseases.

Three hundred gender-matched healthy subjects were also selected as the control group. Controls were healthy Yangzhou residents who accepted annual examination in the physical examination center of Northern Jiangsu People’s Hospital from June 2009 to June 2015. They were chosen randomly from the daily appointment list and were gender matched with the MIEH group. The inclusion criteria for the control subjects were: (1) no personal or family history of hypertension, and (2) a systolic blood pressure (SBP) of < 130 mmHg and a diastolic blood pressure (DBP) of< 85 mmHg. The occurrence of hypertension in one or both biologic parents was considered to be a positive family history of essential hypertension. All study participants were interviewed and then evaluated to identify both personal and medical histories of clinical abnormalities.

Verbal Informed consent, medical history, clinical evaluations and genetic analysis were obtained from all participants involved in the study. The reason for receiving verbal consent is that genetic analysis is used for diagnosis, not for treatment. There was no harm to the patients. The protocol was conducted in accordance with the Helsinki declaration and approved by the ethics committee of the Northern Jiangsu People’s Hospital.

### Data collection

Height and weight were both measured when the subjects had fasted overnight and were wearing only underwear. Body mass index (BMI) was calculated as weight in kilograms divided by height in squared meters (kg/m^2^). Blood pressure was measured by an experienced physician using a mercury column sphygmomanometer according to the World Health Organization (WHO) standardized criteria [20]. The physician was blinded to the study information of the subjects. Systolic and diastolic blood pressure were indicated by the first and fifth Korotkoff sounds, respectively. Three systolic and diastolic blood pressure readings were taken and the mean was used as the blood pressure measurement. The hypertension was defined according to the 2010 Chinese guidelines for the management of hypertension [21]: under the condition of no antihypertensive drugs treatment, the systolic blood pressure is higher than 140 mmHg and/or diastolic blood pressure is higher than 90 mmHg measured three times on different days. After 12-h fast, 4 ml venous blood were drawn from the antecubital vein for the measurement of fasting blood glucose (FBG), total cholesterol (TC), low-density lipoprotein cholesterol (LDL), triglycerides (TG), uric acid (UA), creatinine (CR) and blood urea nitrogen (BUN) by an automatic biochemistry analyzer (Hitach 7600DDP, Japan), using Roche biochemical reaction kits.

### Mitochondrial DNA analysis

Genomic DNA was extracted from peripheral blood using standard protocols [22]. DNA was isolated using Promega Wizard Genomic DNA Purification Kit (Madison, WI, USA). Locations considered the main areas for cardiovascular disease as described previously [12] were screened using oligodeoxynucleotides at 7908-8816 bp. Polymerase chain reaction (PCR) was carried out to amplify mitochondrial tRNA^Lys^ gene using the following primers: forward: 5′-ACGAGTACACCGACTACGGC-3′ and reverse: 5′- TGGGTGGTTGGTGTAAATGA-3′. PCR was performed in 30 μl of the reaction mixture, containing 5.2 μl of PCR Master Mix (Qiagen; Hilden, Germany), 2.5 μl of each primer, 1 μl DNA sample, and 18.8 μl of water. The cycling program for PCR consisted of one cycle of 95 °C for 5 min and then 35 cycles of 95 °C for 30 s, 54 °C for 30 s and 72 °C for 60 s with a full extension cycle of 72 °C for 10 min in a 9700 Thermocycler (Perkin-Elmer Applied Biosystems, Norwalk, USA). Each fragment was purified and subsequently analyzed by direct sequencing with ABI 3730 Sequence Analysis software (Applied Biosystems, Inc., Foster City, CA, USA) using the BigDye Terminator v1.1 kit (ABI Company, Carlsbad, CA, USA), and SeqWeb program GAP(GCG) was used for analysis referring to the updated consensus Cambridge sequence [23]. Pathogenic variants were identified from MitoMap (http://www.mitomap.org/) [24].

### Statistical analysis

Statistical analysis was performed using R and SPSS software (version 16.0; SPSS Inc., Chicago, IL, USA). For comparison of the MIEH group and control group, continuous variables were first tested for normal distribution by Kolmogorov-Smirnov test and then presented in terms of mean ± standard deviation (SD). Discrete variables in the groups were expressed as frequency. Student’s t test and Fisher’s exact t test were used to identify the associations between potential continuous and discrete factors and MIEH respectively. A multiple testing adjusted *P*-value of < 0.05 was considered as statistically significant.

## Results

### Clinical evaluation of baseline characteristics

The general data of study participants is summarized in Table 1. In this study, no significant differences were found in age, gender, or total cholesterol between the two groups. There was a statistical difference for BMI, waist circumference (WC), abdominal circumference (AC), TG, LDL, FBG, UA, CR and BUN (*P* < 0.05) between the two groups.

**Table 1:** Comparison of baseline clinical data between the MIEH and control groups

Subjects	MIEH group	Control group	*p-*value
Gender(M/F)	300(148/152)	300(145/155)	0.870
Age at test(years)	66.85 ± 7.24	65.37 ± 6.76	0.01*
Age at onset (years)	47.46 ± 6.8	NA	
SBP(mmHg)	148.5 ± 19.8	145.6 ± 18.6	0.065
DBP(mmHg)	94.8 ± 8.9	88.4 ± 12.5	< 0.001*
BMI(kg/m^2^)	25.89 ± 2.61	23.55 ± 3.04	< 0.001*
WC (cm)	87.30 ± 10.78	78.08 ± 8.72	< 0.001*
AC (cm)	89.51 ± 10.15	80.98 ± 7.89	< 0.001*
Alcohol, n (%)	72(24)	30(10)	< 0.001*
Current Smoking, n	66(22)	39(13)	0.005*
TG(mmol/L)	1.87 ± 1.22	1.38 ± 0.81	< 0.001*
TC(mmol/L)	4.59 ± 1.90	4.29 ± 1.18	0.021*
LDL(mmol/L)	2.64 ± 1.02	2.03 ± 1.35	< 0.001*
FBG (mmol/L)	5.18 ± 2.19	4.35 ± 0.84	< 0.001*
UA(umol/L)	367.00 ± 127.27	320.38 ± 78.91	< 0.001*
Cr(ummol/L)	103.36 ± 33.71	86.38 ± 30.71	< 0.001*
BUN(mmol/L)	5.75 ± 2.04	4.87 ± 1.78	< 0.001*

Abbreviations: *F* female, *M* male, *SBP* Systolic blood pressure, *DBP* Diastolic blood pressure, *BMI* Body mass index, *WC* waist circumference, *AC* abdomen circumference, *TG* triglyceride, *TC* total cholesterol, *LDL* low-density lipoprotein cholesterol, *FBG* fasting blood glucose, *UA* uric acid, *Cr* creatinine, *BUN* blood urea nitrogen

*: A *P* value < 0.05 was marked by a star

### mtDNA analysis

**Fig. 1:**
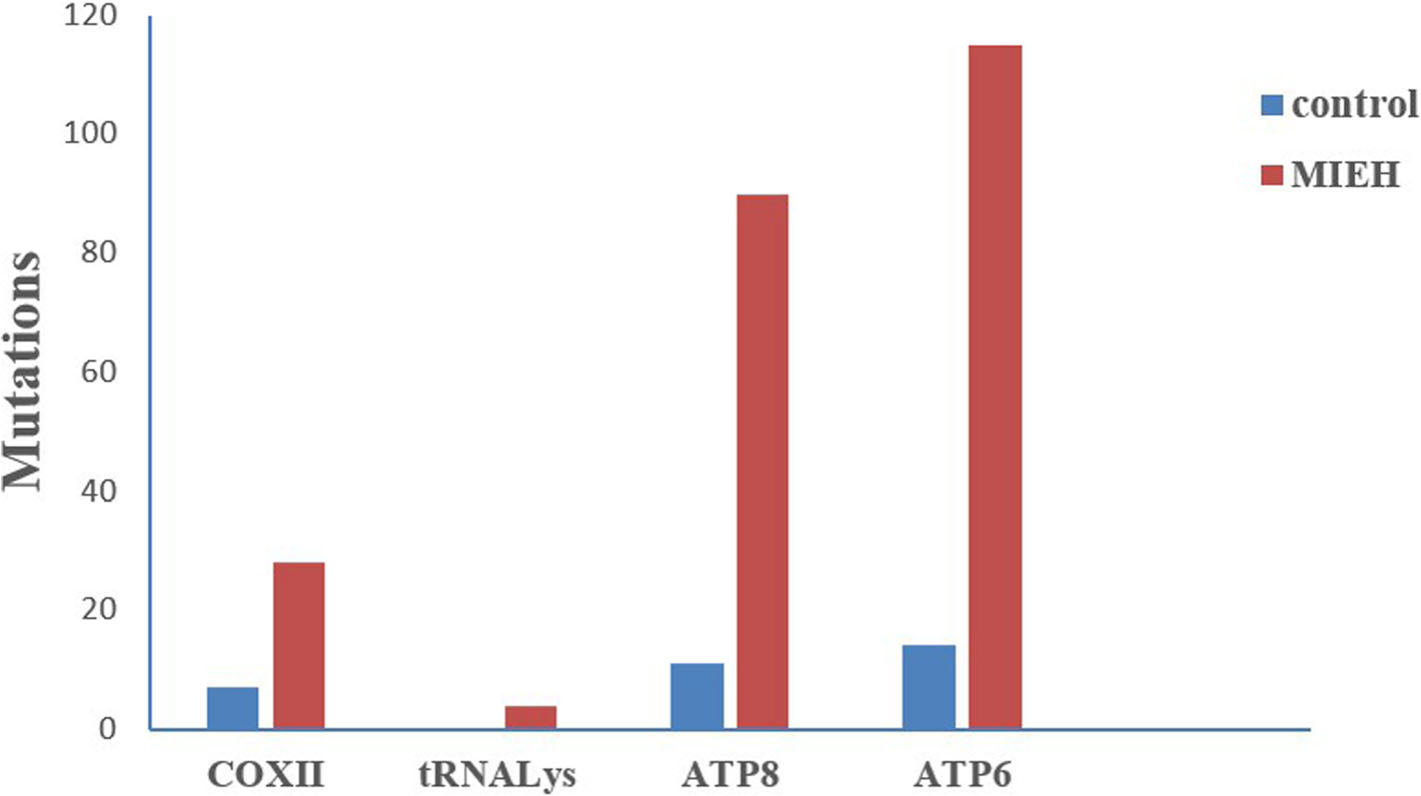
Distribution histogram of the mtDNA sequences in 7908~ 8816

**Fig. 2:**
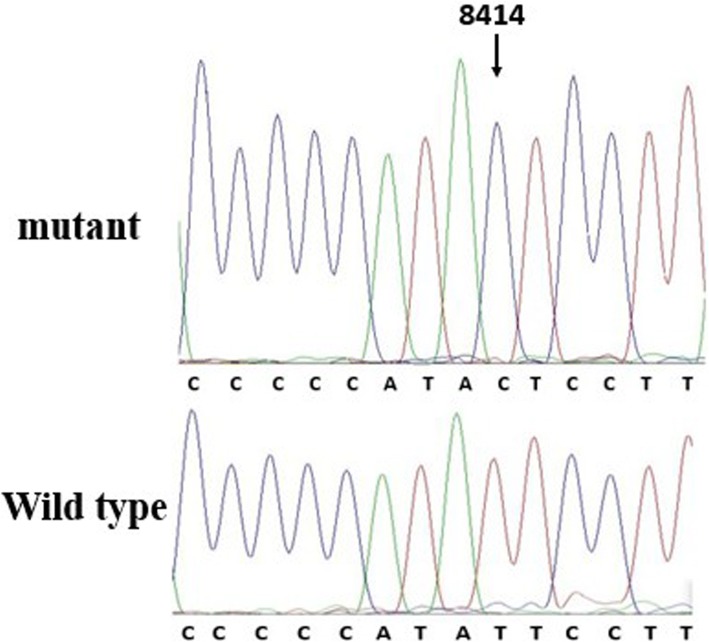
Identification of the m.8414C > T mutation in the mitochondrial ATP8 gene. Arrow indicates the position of the ATP8 gene mutation

Data comparing the frequency of mtDNA variants in the 300 balanced cases and controls are presented in Table 2. The distribution of the number of observed mutations in mtDNA 7908~ 8816 bp for all the participants is shown in Fig. 1. As shown in Table 3, we found a total of 40 mutation sites in the 300 MIEH subjects from the mutation analysis (Additional file 1). The mutations were mainly distributed in the regions of the ATP6 binding site, and the site of highest mutation frequency was m. 8414C > T (Fig. 2).

**Table 2:** Distribution of mtDNA sequence analyses at positions 7908–8816

Gene	Position	Length	Control group (n(%))	MIEH group (n(%))	Fisher’s exact *P* value
COXII	7908–8269	684	7(2.3%)	28(9.3%)	< 0.001
tRNA^Lys^	8295–8364	70	0	4(1.3%)	0.124
ATP8	8366–8572	207	11(3.7%)	90(30%)	< 0.001
ATP6	8527–8816	290	14(4.7%)	115(38%)	< 0.001

**Table 3:** Mutation sites of mtDNA in MIEH individuals and controls

Site of mutation	Gene	Replacement	Number of mutations(n)		Fisher’s exact *P* value	Conservation (H/B/M/X)^a^	Previously reported^b^	Change of Amino acid
			(MIEH)	(Controls)				
8020	COXII	G to A	1	0	1	G/T/C/G	Yes	non-synonymous variant
8027	COXII	G to A	4	2	0.686	G/C/C/A	Yes	non-synonymous variant
8078	COXII	G to A	2	1	1	G/G/A/C	Yes	non-synonymous variant
8149	COXII	A to G	3	1	0.624	A/A/C/T	Yes	non-synonymous variant
8152	COXII	G to A	2	0	0.499	G/A/C/C	Yes	non-synonymous variant
8176	COXII	T to C	2	0	0.499	T/A/C/T	Yes	non-synonymous variant
8200	COXII	T to C	5	1	0.216	T/T/T/C	Yes	non-synonymous variant
8251	COXII	G to A	6	1	0.123	G/T/T/T	Yes	non-synonymous variant
8269	COXII	G to A	2	1	1	G/A/A/C	Yes	non-synonymous variant
8348	tRNA^Lys^	A to G	2	0	0.499	A/T/T/G	Yes	non-synonymous variant
8380	ATP8	T to C	1	0	1	T/T/C/C	No	non-synonymous variant
8392	ATP8	G to A	2	0	0.499	G/A/T/T	Yes	non-synonymous variant
8414	ATP8	C to T	60	7	9.883e-13	C/T/A/T	Yes	non-synonymous variant
8440	ATP8	A to G	1	0	1	A/C/C/T	Yes	non-synonymous variant
8452	ATP8	A to G	1	0	1	A/A/G/T	No	non-synonymous variant
8459	ATP8	A to G	4	1	0.373	A/T/C/G	No	non-synonymous variant
8467	ATP8	C to T	2	0	0.499	C/A/T/T	No	non-synonymous variant
8470	ATP8	A to G	2	0	0.499	A/C/A/T	Yes	non-synonymous variant
8473	ATP8	T to C	4	1	0.373	T/A/A/C	Yes	non-synonymous variant
8557	ATP8	G to A	1	0	1	G/C/G/T	Yes	non-synonymous variant
8563	ATP8	A to G	4	1	0.373	A/T/G/C	Yes	non-synonymous variant
8563	ATP8	A to T	1	0	1	A/T/G/C	No	non-synonymous variant
8584	ATP6	G to A	46	5	5.188e-10	G/T/C/A	Yes	non-synonymous variant
8593	ATP6	A to G	1	0	1	A/C/C/A	No	non-synonymous variant
8654	ATP6	T to C	1	0	1	T/A/A/G	Yes	non-synonymous variant
8656	ATP6	A to G	3	1	0.624	A/G/C/T	No	non-synonymous variant
8684	ATP6	C to T	9	2	0.063	C/G/T/T	Yes	non-synonymous variant
8701	ATP6	A to G	49	6	3.445e-10	A/A/T/A	Yes	non-synonymous variant
8723	ATP6	G to A	1	0	1	G/A/A/T	No	non-synonymous variant
8190	COXII	C to T	1	0	1	C/T/A/A	No	synonymous variant
8343	tRNA^Lys^	A to G	2	0	0.499	A/T/A/C	Yes	synonymous variant
8403	ATP8	T to C	1	0	1	T/A/G/C	No	synonymous variant
8409	ATP8	C to T	3	1	0.624	C/T/A/T	No	synonymous variant
8448	ATP8	T to C	2	0	0.499	T/A/C/G	Yes	synonymous variant
8503	ATP8	T to C	1	0	1	T/T/T/T	Yes	synonymous variant
8604	ATP6	T to C	1	0	1	T/C/A/A	Yes	synonymous variant
8614	ATP6	T to C	1	0	1	T/T/A/T	Yes	synonymous variant
8643	ATP6	C to T	1	0	1	C/C/C/T	No	synonymous variant
8745	ATP6	A to G	1	0	1	A/A/C/G	No	synonymous variant
8749	ATP6	T to C	1	0	1	T/A/T/T	Yes	synonymous variant

^a^H/B/M/X means human/bovine/mouse/xenopus

^b^See http//www.mitomap.org and http://www.genpat.uu.se/mtDB/. Previously reported means the variant was ever reported in a database

**Fig. 3:**
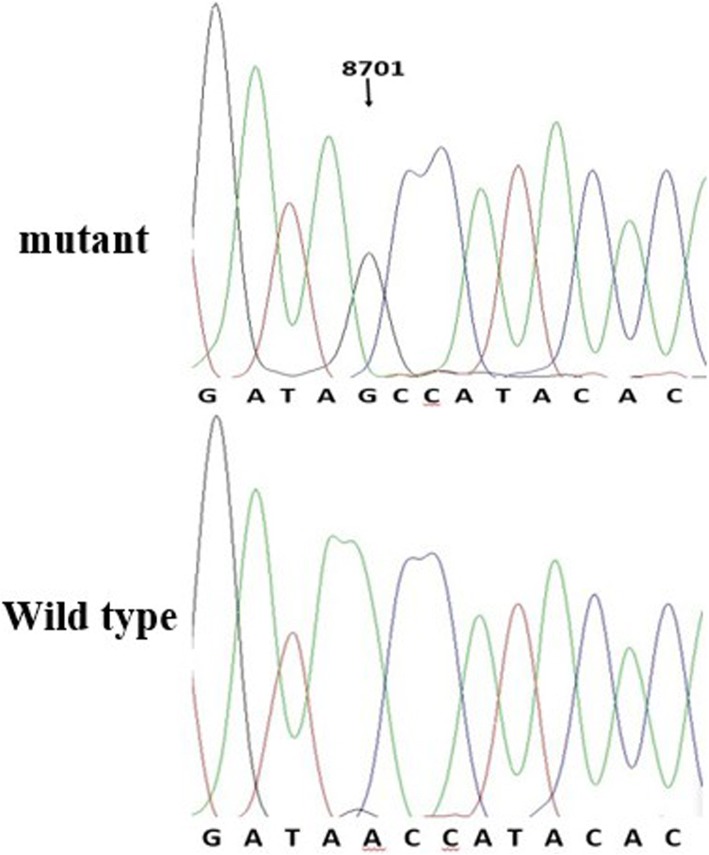
Identification of the m.8701A > G mutation in the mitochondrial ATP6 gene. Arrow indicates the position of the ATP6 gene mutation

**Fig. 4:**
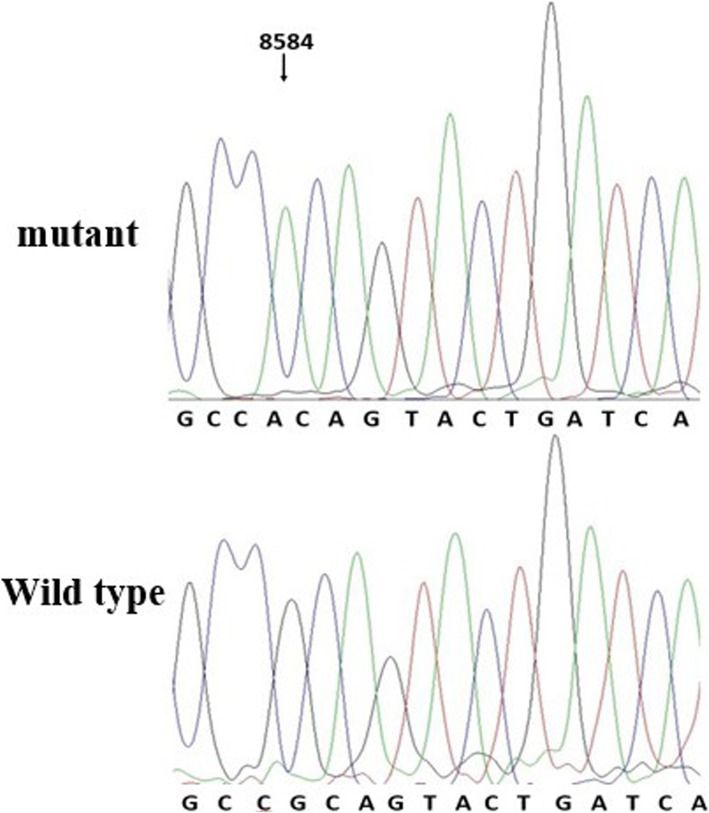
Identification of the m.8584G > A mutation in the mitochondrial ATP6 gene. Arrow indicates the position of the ATP6 gene mutation

These results all showed that the MIEH group had more mtDNA variations in frequency and density than the control group. Three SNPs were significantly (*P* < 0.001) different between the MIEH and the control groups: C8414T (leucine to phenylalanine, belongs to haplogroup D) in ATP8 gene, A8701G in ATP6 gene (threonine to alanine, belongs haplogroup M), and G8584A in ATP6 gene (alanine to threonine) (Figs. 3 and 4).

**Fig. 5:**
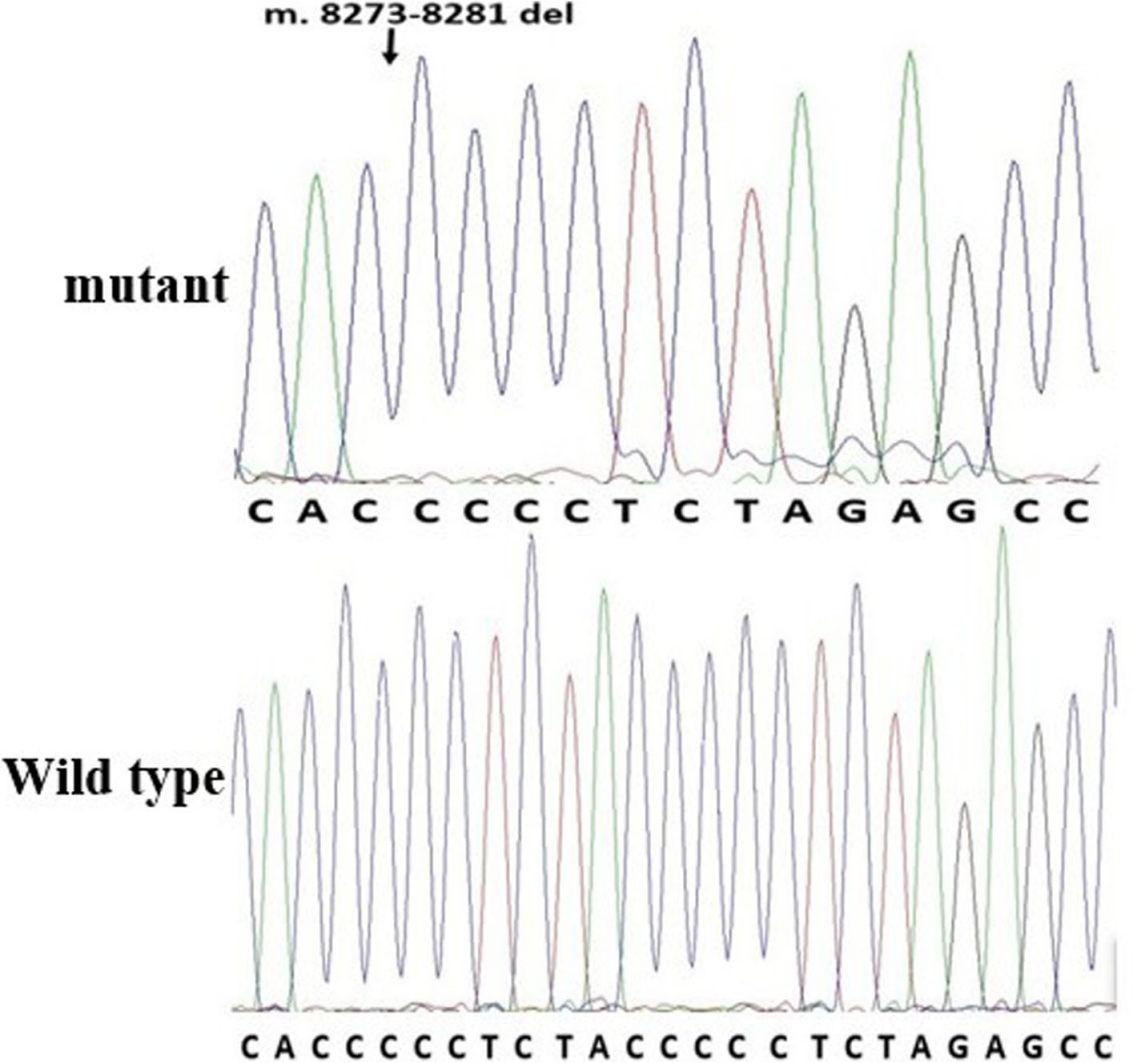
Identification of the m.8273_8281del mutations. Arrow indicates the position of the deletion mutation

Forty different heteroplasmic or homoplasmic mutations were simultaneously identifed in 4 genes: COXII, tRNA^Lys^, ATP8 and ATP 6 gene. We found a total of 38 homoplasmic mutations in 182 MIEH subjects. Two heteroplasmic mutations of m.8563A > T and m.8031C > A were found in 2 MIEH subjects. The MIEH subjects harbored more variants (*P* < 0.01) than the controls with respect to the amino-acid changes and coding sequence variants. Among the MIEH individuals, an intriguing observation was that there were m.8273_8281del mutations in 59 MIEH group patients (Fig. 5). These observations suggested a positive correlation between mtDNA mutation and MIEH.

## Discussion

This study aimed to investigate mtDNA 7908–8816 region mutations in a Chinese population of patients with MIEH. The results showed that the patients in the MIEH group harbored more mtDNA variants than the control group. We simultaneously identified 40 different heteroplasmic or homoplasmic mutations in 4 genes: COXII, tRNA^Lys^, ATP8 and ATP6. As previously reported, most pathogenic mtDNA mutations are in tRNAs [25]. Mutations in protein-encoding genes are frequently associated with ATPase dysfunction [26]. Failure in tRNA metabolism will lead to the deficiency of mitochondrial protein synthesis [27]. Defects in mitochondrial translation consequently result in a respiratory phenotype and a decrease in ATP production,may reduce the production of ROS,and subsequently have the potential role to affect the course of hypertension [28]. The mtDNA variations were mainly distributed in regions of ATP6 binding sites, and the site of highest mutation frequency was m.8414C > T. Three single base pair changes were significantly different between the MIEH and control groups, namely C8414T in ATP8, and A8701G and G8584A in ATP6. It is becoming established that mitochondrial damage and dysfunction are important factors in cardiovascular disease [29]. To our best knowledge, this is one of the first large-scale population-based systematic screens for mitochondrial mutations and their effects on MIEH in the Chinese population. Systematic study of the relationship between disease and mtDNA mutation is important to assist with our understanding of the mechanism of the mutation and its relationship to disease, but is also able to improve diagnosis, prevention and treatment of EH.

Blood glucose, blood lipids, creatinine, urea nitrogen and other biochemical abnormalities are all very closely related to the chance of developing primary hypertension [30]. In order to clarify whether mtDNA affects the biochemical indicators of the MIEH individuals, we compared and analyzed the biochemical abnormalities of the MIEH and normal individuals. Clinical examination and evaluation of all available members in this study suggested that MIEH subjects presented significantly higher values than those of non-maternal members in BMI, WC, AC, TG, LDL, FBG, UA, CR and BUN. In our study, participants with MIEH were overweight or obese compared to participants with normal blood pressure. A normal body weight (BMI 18.5–24.9 kg/m^2^) should be maintained for prevention and management of hypertension [31]. The occurrence and development of MIEH, can involve these factors or these factors might occur as a result of the development of MIEH, which leads to the damage or deterioration of the target organ.

Many studies, including the analysis of maternally transmitted hypertension in a large Han Chinese pedigree, have acknowledged the role of inherited mtDNA mutations in familial MIEH [32, 33]. Here, we undertook mutational analysis of the mitochondrial DNA 7908–8816 region using PCR amplification and then sequence analysis of the PCR fragments. The present experiment showed that there were more mtDNA variations in frequency and density in the MIEH patients than those who were normotensives (NT). Among these mutations, ATP6 is a hotspot for pathogenic mutations associated with MIEH. The occurrence of mtDNA mutations in these genetically unrelated subjects affected by MIEH suggests that mutations may participate in key functional development processes of EH. There are hundreds of mitochondria and thousands of mtDNAs in a mammalian cell and the close proximity of mtDNA within mitochondria with ROS generation sites means that mtDNA is vulnerable to a high level of mutation without an efficient DNA protection and repair system [18].

Mutations of mtDNA may lead to disease, and the significant determinant of their clinical outcomes are likely to correlate with the amount of mutated mtDNA [34]. Some MIEH patients in the study were part of one family branch. Among the MIEH individuals, there was a high mutation frequency and density in mtDNA m.8584G > A and m.8701A > G mutations. Specifically, an amino acid change at the m.8414C > T in the ATP8 gene (leucine to phenylalanine) shows that mtDNA variants may be able to affect the development of hypertension in China [35]. Ancestral variants of mtDNA define population-specific mtDNA lineages or haplogroups. These were first used to trace the origins of different races and allow reconstruction of the migration of humans throughout our ancient history [36]. MtDNA lineages have been shown recently to be more prone to certain disease symptoms, including type-2 diabetes, obesity and atherothrombotic cerebral infarction. Haplogroups can also protect against myocardial infarction and increase lifespan.

MtDNA mutations, including point mutations, deletions, and duplications that affect transcription and translation of mtDNA are implicated in most mitochondrial diseases [37]. Our intriguing observation is that there was a m.8273_8281del mutation in 59 MIEH patients. This 9-bp deletion polymorphism is a phylogenetic marker of studies into evolution trends and population migration [38]. Because of its location, this polymorphism could change either downstream or upstream gene expression. Some crucial structural components of the respiratory chain, such as ATP8, ATP6 are located in the downstream of 9-bp deletion polymorphism [39]. This could affect important respiratory chain structural components, including ATP8 and ATP6 that are located downstream of this polymorphism. If these genes are abnormally expressed they could change oxidative phosphorylation and influence oxidative stress levels [40].

Compared to MIEH patients without mtDNA mutations, the onset time of hypertension for patients with mtDNA mutations was significantly ahead of schedule. This is comparable to the onset time for other Chinese patients with maternally transmitted hypertension [41]. Individuals carrying mtDNA mutations develop hypertension stimulated by environmental factors more easily. Impaired mitochondrial function may contribute increased blood pressure characteristic of aging. Heteroplasmy of mtDNA is strongly associated with hypertension, with the EH mother carrying the gene mutation, and the children with the same mutation more likely to suffer from hypertension. Regarding amino-acid changes and RNAs variants the MIEH subjects harbored more variants than the controls. A possible reason for mtDNA defects pathogenesis in hypertension is that it decreases energy production, increases ROS production, resulting in oxidative stress, disrupting signal transduction, and leading to cardiovascular and renal damage, ultimately initiating hypertension [42, 43]. In consist with previous report [44], mtSNPs may affect the development of hypertension in sporadic Chinese hypertensive subjects. Some specific mtSNP within mitochondria may have potential effect in Chinese hypertensives because of their function. How mitochondrial mtSNPs and/or haplogroups interact synergistically needs to be investigated in further studies. This study was the first step in investigating the role of mitochondria in Chinese hypertensives.

This study has some limitations. One was the small sample size and the single center nature of the study. The conclusions would have been strengthened if the more people had been enrolled. We were not able to control for genetic principal components, which is typically essential in any genomic analysis. Additional limitation was that we were not including people treated with antihypertensives, etc. this would have eliminated quite a large number of people from the analysis.

## Conclusions

In conclusion, our data convincingly demonstrated the possibility of mitochondrial mutations being involved in the pathological process of MIEH. We also identified mitochondrial genetic characteristics in MIEH individuals. Our findings may be generalizable to other China and Asian populations with a similar lifestyle. The investigation of the role of mitochondrial dysfunction in MIEH provides critical implications on the understanding and treatment of this disorder. The present research serves as a solid foundation for further study on the association between MIEH and mitochondrial dysfunction, and the cause and effect relationship in this population.

## Additional file

Additional file 1: Online mtDNA mutations of the MIEH patients. (RAR 3557 kb)

## Abbreviations

AC: Abdominal circumference
BMI: Body mass index
DBP: Diastolic blood pressure
EH: Essential hypertension
MIEH: Maternally inherited essential hypertension
MtDNA: Mitochondrial DNA
SBP: Systolic blood pressure
WC: Waist circumference
